# Öffentliche Gesundheitsdienste in der COVID-19-Pandemie: Strategien und Praktiken in ausgewählten europäischen Nachbarländern

**DOI:** 10.1007/s00103-021-03295-z

**Published:** 2021-03-21

**Authors:** Thomas Gerlinger, Phillip Florian Schmidt, Caspar Lückenbach

**Affiliations:** Fakultät für Gesundheitswissenschaften Arbeitsgruppe „Gesundheitssysteme, Gesundheitspolitik, Gesundheitssoziologie“, Universität Bielefeld, Postfach 10 01 31, 33501 Bielefeld, Deutschland

**Keywords:** Internationaler Vergleich, Öffentliche Gesundheitsdienste in Europa, COVID-19, Prävention, Gesundheitspolitik, International comparison, European public health services, COVID-19, Prevention, Health policy

## Abstract

Die Herausforderungen der COVID-19-Pandemie stoßen in europäischen Gesundheitssystemen auf unterschiedliche institutionelle Strukturen und Handlungstraditionen. Dieser Beitrag befasst sich am Beispiel der öffentlichen Gesundheitsdienste (ÖGD) Schwedens, Frankreichs und Österreichs mit der Frage nach den Gemeinsamkeiten und Unterschieden bei den Maßnahmen zur Pandemiebekämpfung (Stand: November 2020).

Österreich ist das unter den Vergleichsländern am wenigsten, Frankreich das am stärksten von der Pandemie betroffene Land. In allen analysierten Gesundheitssystemen existiert ein Spannungsverhältnis zwischen nationalstaatlichen und regionalen Zuständigkeiten. Frankreichs Gesundheitssystem ist besonders zentralistisch ausgerichtet, das schwedische stark regional und kommunal organisiert. Die Regierungen in den Nationalstaaten sind bestrebt, von Parlamentsentscheidungen unabhängige Kompetenzen zur Pandemieeindämmung zu erhalten. Schweden unterscheidet sich bei der Pandemieeindämmung von Österreich und Frankreich durch eine vornehmlich auf Empfehlung und Appelle statt auf Ge- und Verbote stützende Strategie. Dabei ähneln sich die Handlungssequenzen im Pandemieverlauf und, abgesehen von Schweden, auch die zur Pandemieeindämmung eingesetzten Instrumente. Der Verlauf und die getroffenen Maßnahmen in Österreich und Frankreich zeigen deutliche Parallelen zu denen in Deutschland. Der Schutz besonders vulnerabler Gruppen ist in allen Ländern nur unzureichend gelungen und bleibt eine beständige Herausforderung.

## Abstract (en)

The challenges posed by the COVID-19 pandemic face different institutional structures and traditions of action in the European health systems. This article uses the example of the public health services in Sweden, France and Austria to address the question of the similarities and differences in the measures taken to combat the pandemic (status: November 2020).

Among the countries presented in this article, Austria is the least affected by the pandemic and France is the most affected. In all analysed health systems there is a tension between national and regional responsibilities. France’s healthcare system is particularly centralized, while Sweden’s is strongly regional and municipal. Governments in the nation states are striving to obtain pandemic containment powers independent of parliamentary decisions. Sweden differs from Austria and France in that its pandemic containment strategy is based primarily on recommendations and appeals rather than directives and bans. The sequences of action during the pandemic and, apart from Sweden, the instruments used to contain the pandemic are similar. The course of the pandemic and the measures taken in Austria and France show clear parallels with those in Germany. The protection of particularly vulnerable groups has not been sufficiently successful in all countries and remains a challenge to be met.

## Einleitung

Die COVID-19-Pandemie stellt die europäischen Gesundheitssysteme vor ähnliche Herausforderungen, allerdings stoßen diese in den Nationalstaaten auf bisweilen sehr unterschiedliche institutionelle Strukturen und Handlungstraditionen. Dieser Beitrag geht am Beispiel der öffentlichen Gesundheitsdienste (ÖGD) Schwedens, Frankreichs und Österreichs der Frage nach, welche Gemeinsamkeiten und Unterschiede sich aus internationaler Perspektive bei den Maßnahmen zur Pandemiebekämpfung ergaben. Im Folgenden werden zunächst Grundmerkmale der Organisation des öffentlichen Gesundheitswesens in diesen Ländern und sodann die Hauptmaßnahmen zur Pandemieeindämmung skizziert (Stand: November 2020). Abschließend folgt eine vergleichende Betrachtung, die auch Bezüge zu den Merkmalen und Maßnahmen des ÖGD in Deutschland herstellt.

## Schweden

### Charakteristika des schwedischen Gesundheitssystems

Das staatliche Gesundheitswesen Schwedens ist in einem Mehrebenensystem organisiert. Die Bundesebene ist in Gestalt des *Ministeriums für Gesundheit und Soziale Angelegenheiten (Socialdepartementet)* für die übergeordnete gesundheitspolitische Rahmensetzung sowie einen kleineren Teil der im Wesentlichen steuerbasierten Finanzierung zuständig [1]. Der bedeutendere Teil der Finanzierung und die eigentliche Sicherstellung der ambulanten und stationären Gesundheitsversorgung liegt allerdings im Administrationsbereich der 21 Provinzen und 290 Gemeinden [1]. Der Sektor der Langzeitpflege fällt gänzlich in den Verantwortungsbereich der Gemeinden [1].

Damit ist das schwedische Gesundheitssystem hochgradig regionalisiert mit besonderer kommunaler Verankerung. Diese regionale Gliederung ist handlungsleitend für den ÖGD, also diejenigen Institutionen des Gesundheitssystems, die als Teil des direkten Staatsapparates der Bevölkerungsgesundheit verpflichtet sind. Weitere Merkmale sind die vergleichsweise niedrige Bevölkerungsdichte des Landes und die Konzentration eines erheblichen Teils der Bevölkerung in den urbanen Zentren Mittel- und Südschwedens [2].

Auf nationaler Ebene finden sich die Behörden, die für den Erhalt und die Verbesserung der öffentlichen Gesundheit, die Gesundheitsberichterstattung, aber auch in Teilen für die Krisenvorsorge zuständig sind. Federführend sind hier 2 dem Stockholmer Gesundheitsministerium nachgeordnete Institutionen, deren Aufgabenbereiche sich ergänzen: die *Agentur für Öffentliche Gesundheit (Folkhälsomyndigheten) *und die *Nationale Behörde für Gesundheit und Wohlfahrt (Socialstyrelsen)*. Die dort geplanten bzw. organisatorisch angebundenen Maßnahmen zum Infektionsschutz werden in einem System regionalisierter Verantwortung umgesetzt, das Handlungsspielräumen der teilsouveränen Provinzen und Kommunen Schwedens Rechnung trägt.

Die *Agentur für Öffentliche Gesundheit* ist der zentrale Akteur in Fragen der öffentlichen Gesundheit und trägt in der COVID-19-Pandemie die Gesamtverantwortung für den Schutz der schwedischen Bevölkerung [3]. Ihr obliegen die Sammlung, Systematisierung und Bewertung von Gesundheitsdaten. In der Pandemie ist dieser Teil der epidemiologischen Surveillance-Aufgaben Basis der Beratungsarbeit für die schwedische Regierung und der regionalen Gebietskörperschaften [4]. Ferner entwickelt die Agentur für Öffentliche Gesundheit Leitlinien und Empfehlungen für die Angehörigen der Gesundheitsberufe [3]. Darüber hinaus verfügt die Behörde über eigene Hochsicherheitslaboratorien und plant die landesweiten Laborkapazitäten [3]. Die Agentur für Öffentliche Gesundheit kann als maßgebliche Behörde in der *strategisch-fachlichen Beratung* der schwedischen Regierung im Umgang mit der COVID-19-Pandemie bezeichnet werden.

Die *Nationale Behörde für Gesundheit und Wohlfahrt* ist der zweite wichtige Akteur in der Pandemiebekämpfung auf nationaler Ebene. Die Zuständigkeit dieser Behörde erstreckt sich von der Krisenvorsorge für das gesundheitliche und langzeitpflegerische Versorgungssystem über die Lizensierung des Gesundheitspersonals bis hin zur Erstellung nationaler Krankheitsregister und der Todesursachenstatistik [5]. Im Zuge der COVID-19-Pandemie übernimmt die Nationale Behörde für Gesundheit und Wohlfahrt wichtige beratende und logistische Unterstützungsaufgaben für das Krankenversorgungssystem und die sozialen Dienste. Sie kann als maßgebliche Behörde in der *operativ-koordinativen Bekämpfung* der COVID-19-Pandemie bezeichnet werden. So werden in Zusammenarbeit regionaler Behörden regelmäßig Lageberichte erstellt, die aktuelle und regional differenzierte Analysen über den Bedarf an Schutzkleidung und sonstigen pandemierelevanten Medizinprodukten wie Beatmungsgeräten liefern [6]. Die Behörde koordiniert den Ankauf und die Verteilung solcher Güter in die Regionen und erwirkt ggf. auch eine Umverteilung bereits vorhandenen Materials oder benötigten Personals entsprechend Bedarfsanalysen. Die Regierung hat die Nationale Behörde für Gesundheit und Wohlfahrt mit der landesweiten Koordinierung und Weiterentwicklung der normalerweise regional geplanten Intensivkapazitäten betraut [6].

### Strategien und Maßnahmen zur Pandemieeindämmung in Schweden

Die schwedische Strategie zur Pandemieeindämmung unterscheidet sich auffällig von jenen der meisten anderen europäischen Länder [7, 8]. Zwar verpflichtet sich die schwedische Strategie ebenso der Verlangsamung der Infektionsausbreitung, um das Versorgungssystem nicht zu überlasten, sowie dem Schutz der Risikogruppen. Allerdings gab es in Schweden trotz einiger Verschärfungen Mitte November 2020 insgesamt nur wenig harte Einschnitte in das gesellschaftliche Leben. Zur Eindämmung von SARS-CoV‑2 setzt die schwedische Regierung vor allem auf die Eigenverantwortung der Bürger und appelliert an die Einhaltung der Hygieneempfehlungen sowie die Beachtung des räumlichen Abstands [6].

Maßnahmen, die erheblich in das gesellschaftliche und wirtschaftliche Leben eingreifen, wie Ausgangssperren, die flächendeckende Maskenpflicht, die Schließung von Einzelhandelsgeschäften oder die Schließung aller Bildungseinrichtungen, sind nicht erfolgt. Schweden hat sich mit dem Verweis auf die erwarteten negativen Sekundäreffekte dafür entschieden, Kindertagesstätten und Grundschulen nicht zu schließen [9]. Zu den restriktivsten Maßnahmen zählten das Ende März 2020 verhängte und mit nur wenigen Ausnahmen gültige Verbot von öffentlichen Versammlungen von über 50 Personen, Einreisebeschränkungen, das landesweite Besuchsverbot von Einrichtungen der Langzeitpflege sowie Abstandsregelungen im gastronomischen Gewerbe [10]. Diese Maßnahmen wurden, mit Ausnahme einer kurzen Phase der teilweisen Lockerung im Oktober, im November 2020 vor dem Hintergrund steigender Inzidenzen verschärft. Zunächst befristet bis zu den Weihnachtsfeiertagen galt ein Verbot landesweiter öffentlicher Versammlungen und Veranstaltungen mit mehr als 8 Personen und ein Alkoholverkaufsverbot nach 22:00 Uhr. Besonders die strikte Obergrenze bei öffentlichen Versammlungen kommt einer De-facto-Schließung vieler öffentlicher Freizeit- und Kultureinrichtungen gleich.

Die Eindämmungsstrategie Schwedens ist eine Mischung aus Verhaltensempfehlungen (Hygieneregeln, Social Distancing, Heimarbeit etc.) an die Allgemeinbevölkerung, Ausweitung des Test- und Rückverfolgungsgeschehens, Sicherung der intensivmedizinischen Versorgungskapazitäten und konkreten Restriktionen zum Schutz von Risikogruppen [11]. Die Strategie basiert mit Modifikationen auf nationalen und regionalen Pandemieplänen. Das oftmals als Sonderweg bezeichnete Vorgehen der schwedischen Behörden in der Pandemiebekämpfung ist auch vor dem Hintergrund des Nachhaltigkeitsanspruchs an die Strategie zu sehen: Das Kalkül ist, dass Appelle und Empfehlungen vorbehaltloser und langfristiger von der Bevölkerung akzeptiert und beachtet werden.

Ein Schwerpunkt der schwedischen Strategie im Kampf gegen die COVID-19-Pandemie liegt in der Bereitstellung und Aufrechterhaltung einer bedarfsangemessenen intensivmedizinischen Versorgung. So konnten die intensivmedizinischen Versorgungseinheiten im Zuge der Pandemie verdoppelt und eine Überlastung der Krankenhäuser vermieden werden [6].

### Probleme und Kritik an der Pandemieeindämmung in Schweden

Im Lichte der epidemiologischen Daten kann die schwedische Strategie nicht abschließend beurteilt werden. Die Mortalitätsrate war zwar im September 2020 niedriger als in stark betroffenen südeuropäischen Ländern wie Italien und Spanien, überstieg aber bspw. das deutsche Sterblichkeitsgeschehen um ein 5‑Faches [12]. Aus Analyse der Nationalen Behörde für Gesundheit und Wohlfahrt geht hervor, dass knapp die Hälfte aller an COVID-19 Verstorbenen in Altenpflegeheimen lebte (Erhebung bis 07.09.2020; [13]). Insbesondere im Ballungsgebiet Stockholm ist es nicht gelungen, den Eintrag von SARS-CoV‑2 in die Altenpflegeeinrichtungen zu verhindern.

Im Herbst 2020 stieg die Zahl der Neuinfektionen auch in Schweden wieder erheblich an – hier ist besonders die Großstadt Uppsala betroffen. Die Agentur für Öffentliche Gesundheit hat daraufhin in Abstimmung mit der Bezirksverwaltung lokale Richtlinien zur Eindämmung des Verbreitungsgeschehens erlassen. Diese befristeten und lokal begrenzten Richtlinien fordern unter anderem zur möglichst seltenen Nutzung der öffentlichen Verkehrsmittel auf sowie zum Verzicht auf private Feiern. Derartige über landesweite Empfehlungen hinausgehende Richtlinien sind allerdings nicht sanktionsbewehrt, weil eine Einschränkung des Grundrechts auf Bewegungsfreiheit mit dem Verfassungsrecht kollidiert. Die schwedische Regierung plant daher eine Verfassungsänderung, um der Bundesebene in derartigen Krisenfällen mehr Kompetenzen übertragen zu können. Solch eine Verfassungsänderung mit der Möglichkeit von Grundrechtseinschränkungen in nichtmilitärischen Krisenfällen ist politisch umstritten und würde aufgrund des legislativen Prozederes nicht vor 2021 in Kraft treten können.

## Frankreich

### Charakteristika des französischen Gesundheitssystems

Frankreich hat eine zentralistische Verwaltungstradition, die auch in der Gesundheitspolitik zum Ausdruck kommt [14, 15]. Die 18 Regionen sind keine eigenständigen politischen Gebilde, sondern kaum mehr als die regionalen Repräsentanten des Zentralstaats. Auch Gesundheitsförderung und Prävention werden zentralistisch reguliert ebenso wie die soziale Krankenversicherung, die über weit geringere Handlungsspielräume verfügt als die gesetzliche Krankenversicherung in Deutschland.

Auf zentralstaatlicher Ebene ist die *Nationale Behörde für öffentliche Gesundheit („Agence nationale de santé publique“ – ANSP)* für die Identifikation bevölkerungsbezogener Gesundheitsrisiken sowie für die Entwicklung risikosenkender Maßnahmen und Konzepte zuständig [16]. Unter den auf nationaler Ebene angesiedelten staatlichen Agenturen spielt die *Generaldirektion für Gesundheit („Direction général de la santé“ – DGS)* eine wichtige Rolle. Bei ihr handelt es sich um eine der 5 Direktionen, die im französischen Gesundheitsministerium für die Entwicklung und Formulierung der nationalen Gesundheitspolitik in ihren unterschiedlichen Interventionsbereichen zuständig sind [17]. Die DGS hat die Aufgabe, Ziele und Prioritäten für die öffentliche Gesundheit zu entwickeln sowie dafür Handlungsstrategien und Programme auszuarbeiten. Zu den Handlungsfeldern zählt auch die Bekämpfung von Infektionsrisiken.

Seit einigen Jahren unterliegt das französische Gesundheitssystem einem Prozess der Dezentralisierung [18, 19], der vor allem in der Einrichtung *regionaler Gesundheitsbehörden („Agences régionales de santé“ – ARS)* im Jahre 2010 zum Ausdruck kam [14]. Bei ihnen handelt es sich um regionale Untergliederungen des Zentralstaats. Landesweit gibt es insgesamt 26 dieser regionalen Gesundheitsagenturen [20]. Die ARS sollen die regionale Gesundheitsstrategie in enger Kooperation mit den betroffenen Akteuren (v. a. Städten und Gemeinden, Krankenkassen, Leistungserbringern) gestalten. Dabei sollen sie die traditionellen Grenzen zwischen medizinischer Versorgung, Public-Health-Orientierung und der gesundheitlichen und sozialen Fürsorge, insbesondere für vulnerable Gruppen, überwinden [20, 21]. Die Zuständigkeit der ARS schließt die Krankenhausversorgung, die ambulante Versorgung sowie die Gesundheitsförderung und Prävention ein. Sie schließen mit ihnen Vereinbarungen, verhandeln mit Leistungserbringern, führen die Bedarfsplanung im Krankenhaussektor durch und sind für die Gesundheitsberichterstattung verantwortlich.

Die ARS klären über Krankheitsrisiken auf, entwickeln Konzepte zu gesundheitsförderlichem Verhalten und sind Träger gesundheitlicher Überwachungsaufgaben [21]. Hierzu zählen auch klassische gesundheitspolizeiliche Funktionen wie die Überwachung und Bekämpfung von Infektionsrisiken in einer Region. Auch die Sicherstellung der Versorgung in benachteiligten Regionen zählt zu ihren Aufgaben [22]. Die ARS sind dem Gesundheitsministerium unterstellt und ihre Direktorinnen und Direktoren werden vom Gesundheitsministerium ernannt [23]. Die Agenturen sollen nach zentralstaatlichen Vorgaben, nicht zuletzt zur Ausgabenbegrenzung, Strategien zur Gesundheitssicherung entwickeln und umsetzen und dabei regionale Besonderheiten berücksichtigen [18]. Somit sind sie nicht allein Instrumente für eine Regionalisierung der Gesundheitspolitik, sondern auch für die Durchsetzung zentralstaatlicher Steuerungsziele in den Regionen. Mit ihrem umfassenden Zuständigkeitsbereich handelt es sich bei ihnen um Gebilde, die im deutschen Gesundheitssystem in dieser Form nicht existieren.

### Strategien und Maßnahmen zur Pandemieeindämmung in Frankreich

Frankreich hat die COVID-19-Pandemie von Beginn an im Rahmen einer nationalstaatlichen Strategie bekämpft. Im Zentrum standen die Beschränkung von Kontakten, die rasche Erhöhung der Behandlungskapazitäten für Schwerstkranke, die Rückverfolgung der Kontakte von Infizierten und die Testung von Verdachtsfällen. Das Parlament ermächtigte die Regierung, Bürgerrechte zum Zweck der Bekämpfung eines nationalen Gesundheitsnotstands ohne parlamentarische Zustimmung zeitlich befristet einzuschränken (Etat d’Urgence Sanitaire). Diese Ermächtigung wurde mehrmals verlängert [24]. Nachdem die Regierung zunächst eine zurückhaltende Teststrategie verfolgt hatte und mit diesem Vorgehen heftige Kritik auf sich gezogen hatte, ging sie ab der zweiten Märzhälfte 2020 zu einer Ausweitung und Systematisierung der Tests über. Kurz zuvor war, mit Wirkung vom 16.03.2020, bereits ein weitreichender Lockdown verfügt worden, der neben strengen Kontaktbeschränkungen ein Verbot von größeren Veranstaltungen und die Schließung von Behörden, Bildungs- und Erziehungseinrichtungen sowie von Theatern, Restaurants und Cafés vorsah. Anfang Mai wurden diese Maßnahmen nach einem Rückgang der Infektionszahlen gelockert. Im Herbst 2020 stiegen die Infektionszahlen wieder deutlich an und veranlassten die Regierung erneut zu drastischen Kontaktbeschränkungen, die Ende Oktober in Kraft traten. Es wurde eine weitgehende Ausgangssperre verhängt, während der Personen ihr Haus nur aus triftigem Grund, für eine Stunde am Tag und nur innerhalb eines Radius von bis zu einem Kilometer verlassen durften. Außerdem wurden der Einzelhandel sowie Bars und Restaurants geschlossen. Nach Rückgang der Infektionszahlen wurden diese Bestimmungen wieder vorsichtig gelockert. Ungeachtet dessen gehört Frankreich in Europa zu den am stärksten von der Pandemie betroffenen Ländern.

Die regionalen Gesundheitsbehörden spielen bei der Pandemiebekämpfung eine Schlüsselrolle. Auch auf diesem Handlungsfeld sind sie den Weisungen der Zentralregierung unterworfen. Zugleich sind sie gehalten, sich mit den Kommunen und anderen Funktionsträgern (z. B. Krankenkassen oder Gesundheitseinrichtungen) in der Region abzustimmen, um regional und lokal angemessene Entscheidungen zu treffen. Die ARS nehmen eine Vielzahl von Aufgaben wahr. Dazu zählen die Identifikation von Orten, an denen ein erhöhtes Übertragungsrisiko existiert, und die vorsorgliche Testung von Personen, die sich an diesen Orten aufhalten [25]. Sobald in einer Institution mehrere Infektionsfälle auftreten, führen die ARS-Teams vor Ort z. B. Screeningkampagnen durch und geben den betroffenen Einrichtungen Empfehlungen für den Umgang mit den Risiken. Die ARS organisieren die Verteilung von Schutzausrüstungen und organisieren die Schaffung bzw. Freihaltung von Behandlungskapazitäten für Schwerstkranke. Um möglichst nah am Ort des Geschehens zu sein, betreiben sie in ihrem Zuständigkeitsbereich Zweigstellen. Die Rückverfolgung von Kontakten Infizierter erfolgt durch rund 6500 Mitarbeiterinnen und Mitarbeiter der Krankenversicherung, die von den ARS eine Ad-hoc-Schulung erhalten. Im September 2020 stellte die Regierung Mittel für die Einstellung von weiteren 2000 Mitarbeiterinnen und Mitarbeitern zur Verfügung. Die ARS sind für die Rückverfolgung verantwortlich, wenn Infektionen in einer Einrichtung mit Publikumsverkehr aufgetreten sind [26].

### Probleme und Kritik an der Pandemieeindämmung in Frankreich

Sowohl an der Zentralregierung als auch an den ARS wurde in der französischen Öffentlichkeit zum Teil heftige Kritik geübt. Der Zentralregierung wurde die zunächst zurückhaltende Teststrategie vorgehalten. Zentralstaatliche Beschlüsse zur Schließung von Einrichtungen wurden mitunter als zentralistische Übergriffe wahrgenommen und stießen vereinzelt (z. B. in Marseille) auf heftigen Widerstand in den Regionen. Die ARS hätten sich, so ein häufig vernehmbarer Vorwurf, in der Vergangenheit vor allem an den Sparzielen der Zentralregierung orientiert und seien vielerorts zu einer effektiven Koordination der Pandemiebekämpfung nicht in der Lage [27]. In manchen ARS habe in der Vergangenheit ein Personalabbau stattgefunden, der sich in der Pandemie schmerzlich bemerkbar mache [28]. Bei der Wahrnehmung der Aufgaben trat eine Vielzahl von Problemen auf. Vielerorts gab es wegen mangelnder Laborkapazitäten längere Wartezeiten auf einen Test. Personalmangel und die Verzögerung von Auswertungen erschwerten die Kontaktnachverfolgung. Die Corona-Warn-App hat sich als weitgehend unwirksam erwiesen.

## Österreich

### Charakteristika des österreichischen Gesundheitssystems

Der Öffentliche Gesundheitsdienst (ÖGD) ist im föderalen Mehrebenensystem Österreichs im Wesentlichen auf der Landesebene verankert. Seine Aufgaben werden im Rahmen der mittelbaren Bundesverwaltung auf Landes- und Bezirksebene wahrgenommen [29]. In den letzten Jahren trat die historisch gewachsene föderale Heterogenität von Organisation, Aufgaben und Struktur des ÖGD in den Hintergrund und es gewann die bundeseinheitliche Definition von Aufgaben an Bedeutung [29]. Der ÖGD handelt u. a. als Kontroll- und Aufsichtsorgan in Einrichtungen des Gesundheitssystems und beobachtet mögliche neue Gesundheitsgefahren, die bspw. durch Naturkatastrophen oder neue Infektionskrankheiten entstehen. Weiter führt er Aufsicht über die Ausbildung der nichtärztlichen Gesundheitsberufe [30].

Die Überwachung und Kontrolle von Infektionskrankheiten ist eine der Hauptaufgaben des *Bundesministeriums für Soziales, Gesundheit, Pflege und Konsumentenschutz (BMSGPK)*. Wahrgenommen wird diese in Zusammenarbeit mit den Bundesländern, der österreichischen *Agentur für Gesundheit und Ernährungssicherheit (AGES)* und internationalen Gesundheitsbehörden wie dem Europäischen Zentrum für Seuchenkontrolle (ECDC) oder der Weltgesundheitsorganisation (WHO). Auf Ebene der Bundesländer liegen diesbezüglich die Kompetenzen für den Infektionsschutz und die Infektionsepidemiologie – inklusive des Kontaktpersonenmanagements und der Umgebungsuntersuchung [31]. Auf Bezirksebene sind in den Verwaltungsbehörden Gesundheitsämter eingerichtet, in denen Amtsärztinnen und Amtsärzte hoheitliche Aufgaben wahrnehmen. Auf Gemeindeebene werden Aufgaben des ÖGD durch sog. Gemeindeärztinnen und Gemeindeärzte wahrgenommen, die nach landesrechtlichen Vorgaben tätig sind. Amts- und Gemeindeärztinnen und -ärzten obliegen unter anderem auch sanitätspolizeiliche Maßnahmen beim Auftreten von Infektionskrankheiten. Dazu zählen bspw. Maßnahmen der Datenerhebung, der Desinfektion und – soweit notwendig – auch der Quarantäne bzw. Isolation (Epidemiegesetz § 5 Abs. 1, §§ 7–14 u. § 18).

Meldepflichtige Infektionskrankheiten werden über das Epidemiologische Meldesystem (EMS) erfasst [32]. Für das Monitoring und die Aufbereitung der Daten sind die AGES und die *Gesundheit Österreich GmbH (GÖG)* verantwortlich. Letztere ist ein im Jahr 2006 gegründetes wissenschaftliches Public-Health-Institut, das als führendes Kompetenzzentrum für Bevölkerungsgesundheit, Gesundheitsförderung, Prävention, Versorgungsplanung und Qualität im Gesundheitswesen fungieren soll. Die wichtigste rechtliche Grundlage für die Meldung von Erkrankungen sowie für Regelungen zur Isolierung von Kranken, Krankheitsverdächtigen sowie Ansteckungsverdächtigen bildet das Epidemiegesetz [32]. Zudem wurde 2017 die Einrichtung eines Expertenpools für medizinisches Krisenmanagement an Standorten der AGES beschlossen, um die überregionale Organisation des ÖGD zu stärken, insbesondere zur „raschen Intervention bei hochkontagiösen Erkrankungen“ (LGBl Nr. 60/2017 Art. 12 Abs. 2).

### Strategien und Maßnahmen zur Pandemieeindämmung in Österreich

Nach dem Superspreading-Event (Ereignis, das mit vielen Neuinfektionen einhergeht) in Ischgl, auf das im weiteren Verlauf der Pandemie Hunderte von Infektionen in zahlreichen europäischen Ländern zurückgeführt werden konnten, und aufgrund der im Februar und März 2020 auch in Österreich massiv gestiegenen Infektionszahlen, hat der Nationalrat zum 15.03.2020 das erste *Bundesgesetz betreffend vorläufige Maßnahmen zur Verhinderung der Verbreitung von COVID-19 (COVID-19-Maßnahmengesetz – COVID-19-MG)* beschlossen. Es ermächtigte zunächst nur das Bundesministerium zu weitreichenden Beschränkungen des öffentlichen Lebens und bildete die gesetzliche Grundlage für die Verordnung von Ausgangs- und Versammlungsbeschränkungen, für die Schließung von öffentlichen Orten und Betriebsstätten und zur Mitwirkung der Polizei bei der Kontrolle und Durchsetzung dieser Maßnahmen (BGBl. I Nr. 12/2020).

Diese Konzentration von Kompetenzen beim Bundesministerium wurde teils harsch kritisiert und im Laufe mehrerer Gesetzesänderungen zunehmend abgeschwächt. So wurde eine beratende „Corona-Kommission“ eingerichtet und das Einvernehmen des Hauptausschusses des Nationalrates bei bestimmten Maßnahmen notwendig, bspw. bei einer vollständigen Schließung von Betriebs- und Arbeitsstätten, öffentlicher Räume und der Verhängung von Ausgangsbeschränkungen. In der Ende September 2020 veröffentlichten Fassung können neben dem Bundesministerium auch die Landeshauptleute und die Bezirksverwaltungsbehörden derartige Maßnahmen ergreifen. Lediglich die Maßnahmen zur Ausgangsregelung sind dem Bundesministerium vorbehalten (BGBl. I Nr. 104/2020 COVID-19-MG). Die Kontrolle der Auflagen wurde den Bezirksverwaltungsbehörden übertragen (ebd. § 9) und das Epidemiegesetz entsprechend angepasst (BGBl. I Nr. 104/2020 Epidemiegesetz § 15 Abs. 5 u. § 43a).

Das COVID-19-MG ist zunächst bis zum 31.06.2021 gültig und darf maximal bis zum 31.12.2021 verlängert werden (ebd. § 12). Es ermöglicht somit einerseits der Bundesregierung weitreichende Möglichkeiten zur Einschränkung des öffentlichen Lebens, jedoch haben die Bundesländer und auch die Bezirksverwaltungsbehörden im Verlauf der Pandemie (wieder) an Einfluss gewonnen und können eigene weitergehende Einschränkungen vornehmen und durchsetzen, wenn sie dies für erforderlich halten.

Zur raschen Eindämmung der Pandemie wurden in der zweiten Märzwoche 2020 Reisebeschränkungen eingeführt und kurz darauf das öffentliche und wirtschaftliche Leben in Österreich weitgehend heruntergefahren. Mit Inkrafttreten des COVID-19-MG am 15.03.2020 war das Betreten öffentlicher Orte, wie bspw. Markt- und Kirchenplätze oder Parkanlagen, weitgehend verboten. Einen Tag später wurden auch Gastbetriebe, Universitäten, Hochschulen und Schulen geschlossen und strikte Besuchsverbote sowie Quarantäneregeln in Pflegeheimen eingeführt. Im Verlauf des April wurden dann zusätzlich die Öffnungszeiten noch geöffneter Einzelhandelsgeschäfte eingeschränkt. Durch die im Vergleich zu Deutschland strengen Maßnahmen konnten die Infektionszahlen in Österreich im Frühjahr 2020 binnen 4 Wochen deutlich reduziert werden. So sank die Zahl der täglichen Neuinfektionen von etwa 800 in der letzten Märzwoche auf unter 50 in der ersten Maiwoche [33]. Im Gegensatz zu den strikten Einschränkungen des öffentlichen Lebens war das Testgeschehen teils harscher Kritik ausgesetzt. Neben der als zu gering angesehenen Testkapazität wurden lange Wartezeiten, sowohl bei der Coronahotline 1450 wie auch bei den Testergebnissen, beklagt.

Nach einer schrittweisen Öffnung unter Hygieneauflagen und Maskenpflicht im April und Mai 2020 wurden im Zuge der steigenden Fallzahlen im Juni und Juli wieder sukzessive Einschränkungen vorgenommen. Wie in weiten Teilen Europas stiegen die Infektionen im Herbst weiter an und übertrafen die Zahlen vom Frühjahr teils erheblich. Anfang September führte die Bundesregierung ein Ampelsystem ein, welches das Infektionsgeschehen in 4 Stufen auf Bezirksebene abbildet und zur Einleitung entsprechender Maßnahmen anhalten soll. Die Datenlage wird von einer Kommission aus Expertinnen und Experten sowie Politikerinnen und Politikern wöchentlich analysiert und aktualisiert. Die mit der Ampel verbundenen Maßnahmen sind jedoch nur teilweise auf Bundesebene rechtsverbindlich geregelt und die einzelnen Bundesländer behalten sich eigene Maßnahmen zur Bekämpfung der Pandemie vor. Hier zeigt sich die wie in Deutschland starke Kompetenz der Länderebene auf dem Gebiet des Infektionsschutzes und der öffentlichen Gesundheit. Bundesweit geltende Maßnahmen können nur durch politische Aushandlungsprozesse erreicht werden und stellen lediglich einen gemeinsamen Mindeststandard dar, von dem die Bundesländer und die Bezirksebene abweichen können.

Während die Bundesländer also einen erheblichen Einfluss auf die zu treffenden Maßnahmen haben, obliegen die Ausführung und Kontrolle zuvorderst der Bezirks- und der Gemeindeebene. Anders als in Deutschland, wo die einzelnen Gesundheitsämter über Testungen entscheiden, gilt in Österreich eine nationale Teststrategie des Gesundheitsministeriums, die festlegt, in welchen Fällen Testungen vorgenommen werden. Das Ministerium entscheidet auch über Art und Länge von Quarantänemaßnahmen. Eine Änderung der Teststrategie ermöglicht seit Ende Oktober 2020 nun zum einen die Nutzung von Antigenschnelltests im Rahmen der Kontaktnachverfolgung, zum anderen die Testung symptomatischer Personen beim Hausarzt, sofern die Praxis an dem freiwilligen Programm mit Kostenerstattung über die Sozialversicherung teilnimmt.

Im Laufe des November 2020 wurden zunächst Museen, Theater, Kinos und Restaurants und anschließend auch fast alle Läden erneut geschlossen und der Unterricht wieder auf Distanzlehre umgestellt. Mit der Anfang Dezember 2020 in Kraft getretenen 2. COVID-19-Schutzverordnung galten zwischen 20:00 Uhr und 6:00 Uhr weitgehende Ausgangsbeschränkungen. Im Gegenzug durften Handel und Gastronomie wieder tagsüber öffnen und Schulen den Regelbetrieb wieder aufnehmen. Weiterhin begann Österreich Corona-Massentests, um erneut Kontrolle über das Infektionsgeschehen zu gewinnen.

### Probleme und Kritik an der Pandemieeindämmung in Österreich

Eine Umfrage in Behörden aus dem Frühjahr 2020 deutet darauf hin, dass es bis zu diesem Zeitpunkt nur wenige Probleme beim Umgang mit der Pandemie gab. So gingen die Befragten davon aus, dass die Herausforderungen sehr gut zu bewältigen seien. Als Erfolgsfaktoren wurden interne und externe Strukturveränderungen, z. B. Personalumschichtungen sowie der Netzwerkausbau und eine intensivere Zusammenarbeit mit Ehrenamtlichen und der Privatwirtschaft, benannt [33]. Dies wandelte sich im Herbst jedoch mit dem starken Anstieg der Infektionszahlen auf über 5000 Neuinfektionen pro Tag Ende Oktober und über 8000 am Ende der ersten Novemberwoche [34]. Seit Anfang November zeigte auch das Ampelsystem ein flächendeckend hohes Infektionsrisiko (BMSGPK 2020). Bereits im September und Oktober wurde von zu langen Wartezeiten bei Testergebnissen [35] und Problemen bei der Kontaktnachverfolgung [36–38] berichtet. Zum Ende Oktober 2020 scheint sich die Situation weiter zu verschärfen. So berichten mehrere Medien, dass die Kontaktnachverfolgung mit dem verfügbaren Personal kaum noch zu leisten sei [36–38].

## Vergleichende Betrachtung

### Organisation der Gesundheitsdienste und Verhältnis der Handlungsebenen

Im Vergleich der hier betrachteten Länder wird deutlich, dass die jeweiligen öffentlichen Gesundheitsdienste sehr unterschiedlich organisiert sind. Diese Unterschiede kommen auch in der Bekämpfung der COVID-19-Pandemie zum Tragen. Sie betreffen sowohl die Kompetenzverteilung zwischen nationalstaatlicher und regionaler Handlungsebene als auch die Zuständigkeiten der in die Pandemiebekämpfung einbezogenen Institutionen. Während in Frankreich eine zentralstaatliche Ausrichtung dominiert, besitzen in Schweden die in den Landtagen und Kommunen angesiedelten, für öffentliche Gesundheit verantwortlichen Institutionen ein hohes Maß an Autonomie. Zudem sind im schwedischen Gesundheitssystem Prävention und Krankenversorgung weit stärker miteinander verknüpft als in den anderen Systemen. Auch in Österreich existiert eine starke dezentrale Verantwortung, allerdings sind es im Unterschied zu Schweden hier vor allem die Länder, die über eigenständige Kompetenzen verfügen. Von allen hier betrachteten Ländern weist die Organisation des ÖGD in Österreich die größten Ähnlichkeiten mit Deutschland auf.

In allen analysierten Gesundheitssystemen existiert ein Spannungsverhältnis zwischen nationalstaatlichen und regionalen Zuständigkeiten, aus denen auch politische Konflikte zwischen den Handlungsebenen erwachsen. In Frankreich stößt die zentralstaatliche Entscheidungsgewalt auf Kritik. Zwar sind mit den ARS Einrichtungen geschaffen worden, deren Entscheidungen regionalen Besonderheiten besser Rechnung tragen sollen. Auch deshalb sind bisweilen anzutreffende Behauptungen eines puren Zentralismus ein Zerrbild der Wirklichkeit. Allerdings unterstehen sowohl die regionalen Gesundheitsbehörden als auch die Regionalpräfekten letztlich doch der Pariser Zentralgewalt und sind regionale Entscheidungen gegen deren Willen nicht möglich. Die Ereignisse um den Lockdown in Marseille haben verdeutlicht, welches Konfliktpotenzial diese Konstellation birgt, insbesondere wenn gesundheitspolitische Entscheidungen mit drastischen wirtschaftlichen Konsequenzen einhergehen.

In Österreich stieß die zunächst auf den Bund beschränkte Kompetenz zum Erlass weitreichender Beschränkungen des öffentlichen Lebens auf heftige Kritik. Diese Machtkonzentration wurde in der Folge durch die Einführung von Pflichten zur Koordinierung mit den Ländern und durch die Übertragung außerordentlicher Kompetenzen auf die Landesebene relativiert. In Schweden spielte der Konflikt im föderalen Mehrebenensystem eine vergleichsweise geringe Rolle, weil die Regierung auf Zwangsmittel weitgehend verzichtete und sich überwiegend auf Empfehlungen stützte. Aber auch hier strebt die Regierung eine Verfassungsänderung an, die ihre Kompetenzen im Fall eines nationalen Gesundheitsnotstands erweitern soll.

Bereits zu Beginn der Pandemie waren in Frankreich und Österreich – in ähnlicher Weise wie in Deutschland – die Rechte der Regierung ausgeweitet worden, ohne Zustimmung des Parlaments Bürgerrechte befristet einzuschränken, insbesondere im Hinblick auf die Freizügigkeit. Alle Gesundheitssysteme machten in der Pandemiebekämpfung die Erfahrung, dass eine gesundheitliche Notsituation eher die Stunde der Exekutive und nicht die des Parlaments ist, und überall wurde dies auch heftig kritisiert. Die Motive zur Ausweitung von Regierungskompetenzen – gegenüber regionalen Gebietskörperschaften wie gegenüber dem Parlament – sind doppelter Natur: Zum einen soll sie rasche Reaktionen ermöglichen, zum anderen soll sie möglichst einheitliche Bestimmungen gewährleisten. Letzteres speist sich vor allem aus der Befürchtung, dass eine Vielfalt von Regelungen („Flickenteppich“) Widersprüche und Ungleichbehandlungen hervorbringt, die die Akzeptanz von Einschränkungen in der Bevölkerung mindern könnten. Diese Aspekte spielen auch in Deutschland eine wichtige Rolle. Hier hat sich die Kooperationsbereitschaft zwischen Bund und Ländern schließlich doch als recht groß erwiesen – bei allen Interessen- und Meinungsunterschieden, die im Übrigen weniger aus parteipolitischen Motiven resultieren als aus der unterschiedlichen Betroffenheit von Ländern durch die Pandemie.

### Maßnahmen und Probleme

Die Handlungssequenzen im Pandemieverlauf und die zur Pandemieeindämmung eingesetzten Instrumente waren – bei allen Unterschieden in der Schärfe der Einschnitte und in der Zuständigkeiten und der Kooperation der beteiligten Institutionen – in den hier betrachteten Gesundheitssystemen recht ähnlich und zeigten deutliche Parallelen zu den Ereignissen und Maßnahmen in Deutschland. Dies gilt auch für Schweden, sieht man einmal davon ab, dass die dortige Regierung von Zwangsmaßnahmen nur wenig Gebrauch machte. Auf eine Verschärfung von Maßnahmen bis hin zum Lockdown Mitte März 2020 (außer in Schweden) folgte nach dem Rückgang der Infektionszahlen und Sterbefälle eine Lockerung Anfang Mai, die Ende Oktober erneut von drastischen Einschnitten abgelöst wurde. Im Mittelpunkt standen in den hier betrachteten Gesundheitssystemen – wie auch in Deutschland – die Vermittlung von Informationen zur Kontaktbeschränkung, die Identifikation und Ausschaltung besonderer Infektionsgefahren, die Nachverfolgung der Kontakte von Infizierten, die Beschaffung von Schutzausrüstungen, die Erhöhung von Laborkapazitäten, die Ausweitung der Tests sowie die Schaffung ausreichender intensivmedizinischer Behandlungskapazitäten.

Belastbare Daten über die technische, infrastrukturelle und personelle Infrastruktur der öffentlichen Gesundheitsdienste liegen nicht vor. In allen Gesundheitssystemen traten anfangs Versorgungsengpässe bei Schutzausrüstungen und reduzierte Laborkapazitäten auf. Vor dem Hintergrund steigender Infektionszahlen wurden im Herbst 2020 Laborkapazitäten aufs Neue knapp. In Frankreich wurde über personelle Engpässe bei der Nachverfolgung der Kontakte von Infizierten berichtet. Hier haben sich – ebenso wie im Hinblick auf den ÖGD in Deutschland – die Einsparungen der vergangenen Jahre ausgewirkt. Alle Gesundheitssysteme beklagen überdies einen Mangel an Fachpersonal in der (intensiv-)medizinischen und pflegerischen Versorgung. Verantwortlich ist dafür neben einem ohnehin bestehenden strukturellen Mangel auch der Anstieg von Infektionszahlen beim Gesundheitspersonal. Diese Probleme spielen mit dem Auftreten einer neuen Infektionsdynamik im Herbst 2020 auch in Deutschland eine wachsende Rolle. Engpässe bei der intensivmedizinischen Versorgung Schwerstkranker sind in den hier betrachteten Ländern bisher nur in Frankreich aufgetreten. Neben den geringeren Bettenkapazitäten als in Österreich – und Deutschland – ist dies vor allem auf die außerordentlich hohe Zahl von Infektionen zurückzuführen. Für Österreich finden sich im Unterschied zu den ersten Pandemiemonaten erst mit dem Anstieg der Infektionszahlen im Herbst 2020 Berichte über personelle Engpässe bei der Rückverfolgung der Kontakte von Infizierten.

## Fazit

Eine an quantitativen Indikatoren wie Infektionszahlen oder Sterbefällen orientierte Bewertung des Pandemiemanagements in den verschiedenen Staaten ist kaum möglich. Denn nicht nur Testkapazitäten und Testungspraxis unterscheiden sich von Land zu Land und veränderten sich im Zeitverlauf ebenso die Definition und Erfassung von Todesfällen im Zusammenhang mit der SARS-CoV-2-Infektion. Hinzu kommen Unterschiede in den Bedingungen, die für die Verbreitung des Virus relevant sind, wie Bevölkerungsdichte und Siedlungsstruktur, geografische Lage, transnationale und Binnenmobilität sowie Lebensstandard und Ausprägung sozialer Ungleichheiten. Allerdings lässt sich länderübergreifend mit einiger Bestimmtheit feststellen, dass der Schutz besonders vulnerabler Gruppen in allen Ländern nur unzureichend gelungen ist und eine beständige Herausforderung bleibt. Dies betrifft nicht nur die in der Öffentlichkeit benannten Hochrisikogruppen wie Alte und chronisch Kranke. Darüber hinaus wäre es auch erforderlich, die besondere Situation sozial Benachteiligter zu berücksichtigen und die diversen Bevölkerungsgruppen kultursensibel anzusprechen.

## References

[1] Tikkanen R, Osborn R, Mossialos E, Djordjevis A, Wharton GA (2020) International health care system profiles – Sweden. https://www.commonwealthfund.org/international-health-policy-center/countries/sweden. Zugegriffen: 21. Sept. 2020

[2] Statistics Sweden (2019) 50 largest municipalities, by population. https://www.scb.se/en/finding-statistics/statistics-by-subject-area/population/population-composition/population-statistics/pong/tables-and-graphs/rank-lists-municipalities/swedens-50-largest-municipalities-2020/. Zugegriffen: 20. Okt. 2020 (31 December 2019)

[3] Folkhälsomyndigheten (2020) Our mission. https://www.folkhalsomyndigheten.se/the-public-health-agency-of-sweden/about-us/our-mission. Zugegriffen: 21. Sept. 2020

[4] Folkhälsomyndigheten (2020) Brief facts and organization. https://www.folkhalsomyndigheten.se/the-public-health-agency-of-sweden/about-us/brief-facts-and-organization. Zugegriffen: 21. Sept. 2020

[5] Socialstyrelsen (2020) About the National Board of Health and Welfare. https://www.socialstyrelsen.se/en/about-us. Zugegriffen: 21. Sept. 2020

[6] Socialstyrelsen (2020) The role of the National Board of Health and Welfare during the COVID-19 response in Sweden. https://www.socialstyrelsen.se/en/about-us/emergency-preparedness/the-role-of-the-national-board-of-health-and-welfare-during-the-covid-19-response-in-sweden. Zugegriffen: 21. Sept. 2020

[7] Ludvigsson JF (2020). The first eight months of COVID-19 strategy and the key actions and actors that were lnvolved. Acta Paediatr.

[8] Kavaliunas A, Ocaya P, Mumper J, Lindfeldt I, Kyhlstedt M (2020). Swedish policy analysis for Covid-19. Health Policy Technol.

[9] Folkhälsomyndigheten (2020) Covid-19 in schoolchildren. A comparison between Finland and Sweden. https://www.folkhalsomyndigheten.se/contentassets/c1b78bffbfde4a7899eb0d8ffdb57b09/covid-19-school-aged-children.pdf. Zugegriffen: 21. Sept. 2020

[10] Krisinformation (2020) Restrictions and prohibitions. https://www.krisinformation.se/en/hazards-and-risks/disasters-and-incidents/2020/official-information-on-the-new-coronavirus/restriktioner-och-forbud. Zugegriffen: 21. Sept. 2020

[11] Lindström M (2020). The COVID-19 pandemic and the Swedish strategy. Epidemiology and postmodernism. SSM Popul Health.

[12] Johns Hopkins University (2020) Mortality analyses. https://coronavirus.jhu.edu/data/mortality. Zugegriffen: 21. Sept. 2020

[13] Socialstyrelsen (2020) Analys av det tillfälliga förbudet mot besök inom särskilda boendeformer för äldre. https://www.socialstyrelsen.se/globalassets/sharepoint-dokument/artikelkatalog/ovrigt/2020-9-6902.pdf. Zugegriffen: 21. Sept. 2020

[14] Palier B (2017). La réforme des systèmes de santé.

[15] Bouckaert G, Galli D, Kuhlmann S, Reiter R, Van Hecke S (2020). European coronationalism? A hot spot governing a pandemic crisis. Public Admin Rev.

[16] Bourdillon F (2015). Rapport de prefiguration.

[17] Ministère des solidarités et de la santé (2019) Organisation de la direction générale de la santé (DGS). https://solidarites-sante.gouv.fr/ministere/organisation/organisation-des-directions-et-services/article/organisation-de-la-direction-generale-de-la-sante-dgs. Zugegriffen: 1. Nov. 2020

[18] Palier B, Davesne A, Pavolini E, Guillèn AM (2013). France: squaring the health spending circle?. Health care systems in Europe under austerity: institutional reforms and performance.

[19] Bergeron H, Castel P (2015). Sociologie politique de la santé.

[20] Chevreul K, Berg Brigham K, Durand-Zaleski I, Hernández-Quevedo C (2015). France: health system review. Health Syst Transit.

[21] Hassenteufel P, Bras PL, de Pouvourville G, Tabuteau D (2009). Le rôle de l’État dans la régulation de l’assurance maladie. Traité d’économie et de gestion de la santé.

[22] Hassenteufel P, Schweyer FX, Gerlinger T, Henkel R, Lückenbach C, Reiter R (2019). The role of professional groups in policy change: physician’s organizations and the issue of local medical provision shortages in France and Germany. Eur Policy Anal.

[23] Brunn M, Hassenteufel P (2018). Frankreich: Gesundheitspolitik weiter „en marche“?. GuS.

[24] Darame M (2020) Etat d’urgence sanitaire: le Parlement devant le fait accompli. https://www.lemonde.fr/politique/article/2020/10/23/etat-d-urgence-sanitaire-le-parlement-devant-le-fait-accompli_6057089_823448.html. Zugegriffen: 31. Okt. 2020 (Le Monde, 23 octobre 2020)

[25] Service public (2020) Test de dépistage: quelles sont les personnes prioritaires? https://www.service-public.fr/particuliers/actualites/A14296. Zugegriffen: 1. Nov. 2020

[26] Gandre C, Or Z (2020) COVID-19 health system response monitor: France. https://www.covid19healthsystem.org/countries/france/countrypage.aspx. Zugegriffen: 1. Nov. 2020 (last modified: 07/10/2020)

[27] Bergeron H (2020) Coronavirus: pourquoi les Agences Régionales de Santé sont-elles sous le feu des critiques ? https://www.sudouest.fr/2020/04/28/coronavirus-pourquoi-les-agences-regionales-de-sante-sont-elles-sous-le-feu-des-critiques-7444731-4696.php. Zugegriffen: 1. Nov. 2020

[28] Jarjaille I (2020) Covid-19: les agences régionales de santé sous le feu des critiques. https://www.lagazettedescommunes.com/676743/covid-19-les-agences-regionales-de-sante-sous-le-feu-des-critiques/. Zugegriffen: 1. Nov. 2020

[29] Bundesministerium für Gesundheit (2010). Handbuch ÖGD. Handbuch für den Öffentlichen Gesundheitsdienst in Österreich.

[30] Bundesministerium Soziales, Gesundheit, Pflege und Konsumentenschutz [BMSGPK] (2019a) Öffentlicher Gesundheitsdienst (ÖGD). https://www.sozialministerium.at/Themen/Gesundheit/Oeffentlicher-Gesundheitsdienst-. Zugegriffen: 26. Okt. 2020

[31] Öffentliches Gesundheitsportal Österreichs (2018) Gesundheitsverwaltung der Bundesländer. https://www.gesundheit.gv.at/gesundheitsleistungen/institutionen/landessanitaetsdirektion. Zugegriffen: 26. Okt. 2020

[32] Bundesministerium Soziales, Gesundheit, Pflege und Konsumentenschutz [BMSGPK] (2019b) Rechtliche Grundlagen und Meldung übertragbarer Krankheiten. https://www.sozialministerium.at/Themen/Gesundheit/Uebertragbare-Krankheiten/Rechtliches.html. Zugegriffen: 26. Okt. 2020

[33] Schomaker RM, Ruf V, Pöhler J, Bauer MW (2020) Betroffenheit und Reaktionen der österreichischen Kommunen in der COVID-19 Pandemie, Working Paper Series No.6. www.fh-kaernten.at/unserstudienangebot/wirtschaft-management/forschung/working-paper-series/. Zugegriffen: 29. Okt. 2020

[34] Johns Hopkins University (2020) COVID-19 dashboard. https://www.arcgis.com/apps/opsdashboard/index.html#/bda7594740fd40299423467b48e9ecf6. Zugegriffen: 29. Okt. 2020 (by the Center for Systems Science and Engineering (CSSE) at Johns Hopkins University (JHU))

[35] Der Standard (2020) Länder beklagen, dass Contact-Tracing nicht funktioniert – Kurz will Nachbesserungen von Wien. https://www.derstandard.de/story/2000120273196/laender-beklagen-dass-contact-tracing-nicht-funktioniert. Zugegriffen: 29. Okt. 2020

[36] Die Presse (2020) Contact Tracing stößt an Grenzen: „Zuwachs an Fällen kaum mehr abzuarbeiten“. https://www.diepresse.com/5887183/contact-tracing-stosst-an-grenzen-zuwachs-an-fallen-kaum-mehr-abzuarbeiten. Zugegriffen: 29. Okt. 2020

[37] Kurier (2020a) Contact Tracing in der Stadt Salzburg vorübergehend im Notbetrieb. https://kurier.at/chronik/oesterreich/coronavirus-contact-tracing-in-der-stadt-salzburg-im-notbetrieb/401078802. Zugegriffen: 29. Okt. 2020

[38] Kurier (2020b) Immer höhere Fallzahlen im Westen. Contact Tracing in Salzburg im Notbetrieb. https://kurier.at/chronik/oesterreich/who-ortet-pandemie-muedigkeit-duerfen-nicht-aufgeben-anti-corona-demo-in-wien/401077806. Zugegriffen: 29. Okt. 2020

